# Supplementary material for: The causal relationship between the human gut microbiota and pyogenic arthritis: a Mendelian randomization study
Source: Front Cell Infect Microbiol. 2024 Nov 26;14:1452480. doi: 10.3389/fcimb.2024.1452480 (PMC11629706; doi:10.3389/fcimb.2024.1452480)
Supplement: Supplementary file 1 [file DataSheet1.zip › mendelian test/ebi-a-GCST90027572.csv_forest.pdf]

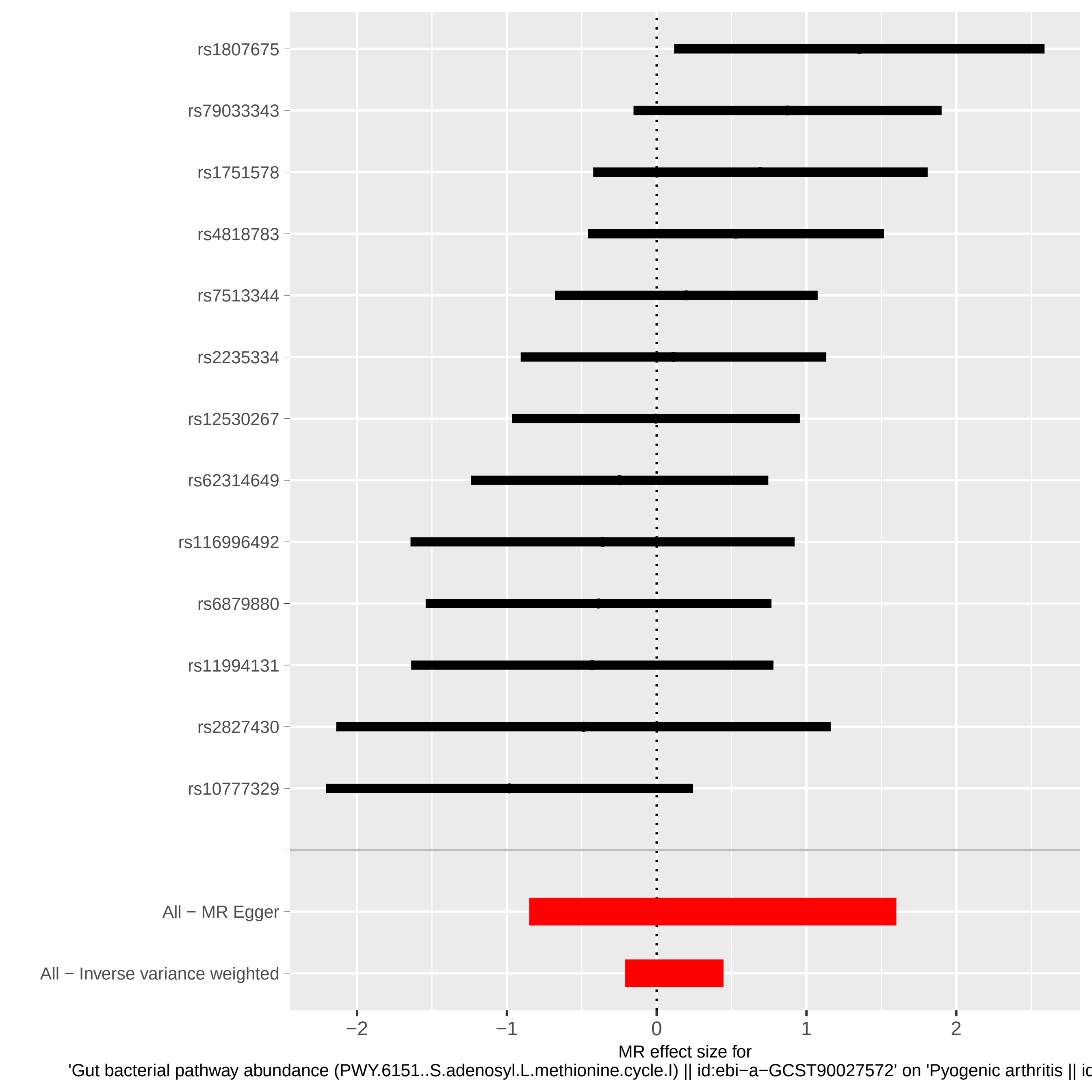
rs1807675
rs79033343
rs1751578
rs4818783
rs7513344
rs2235334
rs12530267
rs62314649
rs116996492
rs6879880
rs11994131
rs2827430
rs10777329
All − MR Egger
All − Inverse variance weighted
−2
−1
0
1
2
MR effect size for
'Gut bacterial pathway abundance (PWY.6151..S.adenosyl.L.methionine.cycle.I) || id:ebi−a−GCST90027572' on 'Pyogenic arthritis || id
